# Regional patterns of grey matter atrophy and magnetisation transfer ratio abnormalities in multiple sclerosis clinical subgroups: A voxel-based analysis study

**DOI:** 10.1177/1352458514546513

**Published:** 2015-04

**Authors:** Shahrukh Mallik, Nils Muhlert, Rebecca S Samson, Varun Sethi, Claudia AM Wheeler-Kingshott, David H Miller, Declan T Chard

**Affiliations:** NMR Research Unit, University College London, UK; NMR Research Unit, University College London, UK/School of Psychology: Cardiff University, UK; NMR Research Unit, University College London, UK; NMR Research Unit, University College London, UK; NMR Research Unit, University College London, UK; NMR Research Unit, University College London, UK; NMR Research Unit, University College London, UK/National Institute for Health Research, University College London Hospitals, UK

**Keywords:** Multiple sclerosis, grey matter, magnetisation transfer ratio, atrophy, voxel based analysis, demyelination

## Abstract

**Background:**

In multiple sclerosis (MS), demyelination and neuro-axonal loss occur in the brain grey matter (GM). We used magnetic resonance imaging (MRI) measures of GM magnetisation transfer ratio (MTR) and volume to assess the regional localisation of reduced MTR (reflecting demyelination) and atrophy (reflecting neuro-axonal loss) in relapsing–remitting MS (RRMS), secondary progressive MS (SPMS) and primary progressive MS (PPMS).

**Methods:**

A total of 98 people with MS (51 RRMS, 28 SPMS, 19 PPMS) and 29 controls had T1-weighted volumetric and magnetisation transfer scans. SPM8 was used to undertake voxel-based analysis (VBA) of GM tissue volumes and MTR. MS subgroups were compared with controls, adjusting for age and gender. A voxel-by-voxel basis correlation analysis between MTR and volume within each subject group was performed, using biological parametric mapping.

**Results:**

MTR reduction was more extensive than atrophy. RRMS and SPMS patients showed proportionately more atrophy in the deep GM. SPMS and PPMS patients showed proportionately greater cortical MTR reduction. RRMS patients demonstrated the most correlation of MTR reduction and atrophy in deep GM. In SPMS and PPMS patients, there was less extensive correlation.

**Conclusions:**

These results suggest that in the deep GM of RRMS patients, demyelination and neuro-axonal loss may be linked, while in SPMS and PPMS patients, neuro-axonal loss and demyelination may occur mostly independently.

## Introduction

In multiple sclerosis (MS), grey matter (GM) demyelination and neuro-axonal loss are all recognised histopathological features.^1^ Cortical demyelination can be seen in the earliest clinical stages of MS,^2^ and may be extensive in progressive forms of the disease.^3,4^ Similarly, GM neuro-axonal loss, especially in the deep GM structures, can be seen early in MS,^5^ and is evident in the cortex later in the course of the disease.^6–8^

It is not known how closely GM demyelination and neuro-axonal loss are related. One histopathological study using material from 22 people with MS (clinical sub-types were not specified) found no clear association between local cortical demyelination and cortical thickness, although neuronal densities were reduced in leucocortical lesions.^6^ More recently, evidence has emerged that meningeal inflammation is linked with both cortical demyelination,^8–10^ and neuro-axonal loss.^7^ Such meningeal inflammation appears to be particularly apparent in secondary progressive (SPMS) and primary progressive (PPMS) subgroups.^8–10^ In spite of these observations, it is uncertain to what extent GM demyelination and neuro-axonal loss are spatially co-localised and whether or not they are caused by a common pathological process. It is also not known if links (if present) between local demyelination and neuro-axonal loss occur in all MS subtypes.

Brain GM atrophy, which is associated with neuro-axonal loss,^11^ can be detected using magnetic resonance imaging (MRI), and appears to have a greater impact on clinical disability in the long-term than white matter (WM) lesion accrual.^12–14^ However, GM atrophy is not uniform throughout the brain, with some regions more frequently affected than others,^15,16^ and this may vary between different MS phenotypes.^17^ Magnetisation transfer (MT) imaging provides insight into intrinsic structural tissue abnormalities. In WM, the MT ratio (MTR) is reduced in the presence of demyelination, and to a lesser degree axonal loss,^18^ and in the cortex has also been shown to reflect predominantly demyelination.^19^ Abnormal GM MTR has been shown even in the earliest stages of MS, correlating with disease duration,^20^ clinical disability^21^ and cognitive impairment.^22^

Voxel-based analysis (VBA) is a fully automated technique which allows whole-brain comparisons to be made between subject groups on a voxel-wise basis, via spatial transformation of images to a normalised template. This method differs from region of interest (ROI) and histogram-based methods in that it allows unbiased assessment of the whole brain, detecting region specific changes between subject groups without first having to define the location and shape of the regions to be assessed.^23,24^ VBA methods allow the detection of significant regional effects within one group compared to another, identifying regions where the majority of participants in one group significantly differ from another group. Voxel-based morphometry (VBM) specifically allows the volume of GM to be compared between subject groups at a voxel-by-voxel level, and is used to quantify atrophy.^25^ Previous voxel-based studies have demonstrated regional MTR reductions in some areas in relapsing–remitting MS (RRMS) (insula and lenticular nuclei bilaterally, as well as the left posterior cingulate cortex, and the right orbitofrontal cortex).^26^ In one study of people with PPMS, MTR changes were found to co-localise with atrophy in small GM regions,^27^ but MTR reductions were also seen in areas without GM atrophy, and in the majority of cases co-localisation in GM was not observed.

To date, no studies have systematically assessed regional GM atrophy and MTR abnormalities across RRMS, PPMS and SPMS patients. In this study we undertook VBA of brain GM atrophy and MTR in a relatively large cohort of people with MS including all three subgroups. Using VBA to identify regions of the brain that are consistently affected in each group, we sought to answer the following questions:

1. Does atrophy, suggesting neuro-axonal loss, demonstrate different spatial patterns of cortical and deep GM involvement in different clinical subgroups of MS?
2. Does MTR reduction, suggesting demyelination, demonstrate different spatial patterns of cortical and deep GM involvement in different clinical subgroups of MS?
3. To what extent do atrophy and MTR reduction occur in the same regions in the cortical and deep GM of different clinical subgroups of MS?

## Methods

### Subjects

We recruited people with clinically definite MS^28^ who had not experienced a relapse or received corticosteroids in the four weeks prior to the study. We also recruited healthy controls with no history of neurological or psychiatric disorders. All participants gave informed consent before taking part in this study. This study was approved by the local institutional research ethics committee. All participants had a clinical assessment to confirm the clinical subtype of MS, disease duration, and disability as measured using the Expanded Disability Status Scale (EDSS).

### MRI acquisition

Images were acquired using a 3T Philips Achieva scanner (Philips Healthcare, Best, The Netherlands) with a 32-channel head coil and multi-transmit technology. T1-weighted (T1w) volumetric and MT images were obtained sagittally, with a field-of view of 256×256×180 mm^3^ and 1 mm isotropic resolution with the following parameters: T1w volumes, using a 3D inversion-prepared (TI=824 ms) gradient echo [Fast Field Echo (FFE)] sequence [Repetition Time (TR)=6.9ms; Echo Time (TE)=3.1ms]; flip angle (α)=8°; MTR data, using a 3D slab selective spoiled gradient echo (FFE) sequence with two echoes (TR=6.4 ms, TE1/TE2=2.7/4.3 ms, α=9°) with and without sinc-Gaussian shaped MT saturating pulses of nominal α=360°, offset frequency 1 kHz, duration 16 ms applied prior to the excitation pulse.

### Image analysis

NiftyReg^29^ was used to affine register the MT-on and MT-off images to the T1w volume. MTR maps were calculated using the equation

MTR=(MT-off–MT-on)/MT-off

WM lesion-filled T1w images^30^ were segmented using the ‘New Segment’ function in SPM8 (UCL Wellcome Trust Centre for Neuroimaging, London, UK). The resulting GM tissue probability maps were all checked for accuracy and then used in two ways. First, they were binarised using a conservative 90% threshold, and overlaid on the MTR map to define GM regions to be included in the subsequent VBM analysis. The binarised GM volumes for each individual subject were divided by their respective intracranial volumes (ICVs), calculated by summing the GM, WM and Cerebrospinal Fluid (CSF) volumes, to calculate the GM fraction (GMf). Second, the probability maps were used to create a custom diffeomorphic anatomical registration using exponentiated Lie algebra (DARTEL) template using SPM8.^31^ Each participant’s GM probability map was registered to this template using a non-linear transformation, and then affine transformed into Montreal Neurological Institute (MNI) space with sinc interpolation, before being smoothed with an 8 mm full width half maximum (FWHM) Gaussian smoothing kernel. Each participant’s segmented GM MTR map was then transformed to MNI space via the DARTEL template using the same transformations in a single step with sinc interpolations. The MTR maps were then smoothed using an 8 mm FWHM Gaussian smoothing kernel. We calculated the global mean GM MTR value for the segmented MTR maps using the ‘fslstats’ command from the FSL suite (FMRIB Software Library, Oxford).^32^ Also using ‘fslstats’, we calculated mean voxel counts for the cortical and deep GM compartments for each group, from the thresholded (90%) T1w GM segmentations.

### Statistical analysis

Using Stata 13.1 (Stata Corporation, College Station, Texas, USA), comparison of means between the subject groups was performed using a multiple regression for each response variable (age, duration and global GM MTR) regressed on three group indicators (RR, PP, SP) with controls as the reference group. Using this model, joint tests were performed with the null hypothesis that all groups demonstrated the same mean; if this null hypothesis was rejected, individual group comparisons were performed from the same model estimates. In the case of global GM MTR, adjustment was made for age, duration and gender which were entered as covariates. Volumetric and MTR VBA was performed using SPM8. Areas of abnormality were considered statistically significant at an alpha threshold of *p*=0.05, corrected for multiple comparisons using Gaussian random field theory (family wise error (FWE)) at voxel level. Age and gender were included as covariates in the general linear models. We also performed analysis at *p*=0.001 uncorrected, for comparison. We used the Wake Forest University (WFU) biological parametric mapping (BPM beta version 1.5d) toolbox for SPM5 to perform a voxel-by-voxel correlation analysis of MTR and volume within each clinical subgroup, at FWE (*p*=0.05).^33^

### Identification and quantification of affected voxels

When comparing each of the three MS clinical subgroups with controls, and comparing the clinical subgroups with each other, we used MRIcron^34^ to (a) label regions of consistent MTR reduction, (b) label regions of consistent atrophy, (c) label regions where MTR reduction and atrophy co-localised, and (d) count the number of voxels affected by each pathological process in each region. This was done by overlaying the images of significant clusters from SPM8 onto the Automated Anatomical Labeling (AAL) atlas.^35^ The same method was used to identify voxels demonstrating correlation between MTR and volume in the GM.

## Results

### Participant characteristics

A total of 98 patients and 29 healthy controls were included in the study. Of the patients, 51 had RRMS, 28 had SPMS, and 19 had PPMS. Participant characteristics are summarised in Table 1. The joint tests from a single model for the null hypothesis that all subject groups had the same mean age and duration were rejected (*p*<0.0001). Overall, all MS subgroups were older than controls (*p*<0.001). People with RRMS were significantly younger than people with SPMS (*p*<0.001) and PPMS (*p*<0.001). There was no significant difference in age between the SPMS and PPMS groups (*p*=0.642). The SPMS group had a significantly longer mean disease duration than either PPMS (*p*=0.001), or RRMS (*p*<0.001). There was no significant difference in mean duration between RRMS and PPMS (*p*=0.348). The joint test from a single model for the null hypothesis that all subject groups had the same mean global GM MTR, when adjusted for age, duration and gender was rejected (*p*<0.0001). Adjusting for age, duration and gender, there was a significant difference in the global GM MTR between all MS subgroups and controls (*p*<0.001), RRMS and SPMS (*p*<0.001), SPMS and PPMS (*p*<0.001), but no significant difference between RRMS and PPMS (*p*=0.104).

**Table 1.** Participant characteristics.

	RR	SP	PP	Controls
***n* (males)**	51 (19)	28 (7)	19 (8)	29 (13)
**Mean age (years)**	42.35	53.43	52.05	36.97
**SD**	9.84	7.73	9.48	12.04
**Range**	21–64	36–65	27–65	22–63
**Mean duration (years)**	11.85	22.73	13.53	–
**SD**	9.09	11.16	7.97	–
**Range**	0.5–45.0	7.5–47.83	2.5–27.25	–
**Median EDSS**	2.0	6.5	6.0	–
**Range**	1.0–7.0	4.0–8.5	1.5–6.5	–
**Use of DMT**	40	13	3	–
**Current use**	29	7	1	–
**Previous use**	11	6	2	–
**Mean GM MTR**	30.74	29.78	30.23	31.86
**SD**	1.211	1.141	1.498	0.566
**Mean cortical voxels**	538,477	499,893	546,057	560,602
**SD**	52,443	56,478	61,745	55,194
**Mean deep GM voxels**	23,446	21,927	24,840	26,353
**SD**	3025	2943	2680	2599

DMT: disease modifying therapy; EDSS: Expanded Disability Status Score; GM: grey matter; GMf: grey matter fraction; MTR: magnetisation transfer ratio; PP: Primary progressive; RR: Relapsing–remitting; SD: standard deviation; SP: secondary progressive.

### Clinical subgroups compared to controls

A summary of results can be seen in Table 2, showing voxel counts for MTR reduction, and atrophy, in the total GM, in the cortex, in the deep GM, for the different clinical subgroups in MS compared with healthy controls.

**Table 2.** Voxel-based analysis (VBA) significant voxel counts (compared with controls) by clinical subgroup and location (family wise error (FWE) 0.05).

	Overall MTR reduction			Overall atrophy		
	RR	SP	PP	RR	SP	PP
**Total**	3615	13109	318	5026	1538	0
**Cortex**	399	9320	318	206	117	0
**Deep GM**	3216	3789	0	4820	1421	0

GM: grey matter; MTR: magnetisation transfer ratio; PP: primary progressive; RR: relapsing–remitting; SP: secondary progressive.

#### MTR reduction

Significant regional MTR reductions were seen in the GM of all clinical subgroups compared to controls, but were more extensive in SPMS (13,109 voxels) than in RRMS (3615 voxels) and PPMS (3825 voxels). The absolute numbers of voxels demonstrating MTR reduction was more in the cortex than in the deep GM in PPMS and SPMS, while in RRMS the deep GM areas were proportionately more affected. The precise regions of cortical and deep GM MTR reduction for each clinical subgroup, compared to controls, are summarised in Supplementary Material, Appendices 1 and 2.

#### Atrophy

Compared to controls, the number of voxels demonstrating regional atrophy was more extensive in RRMS (5026 voxels) and SPMS (1538 voxels) than in the PPMS group (no significant regional atrophy compared to controls). Deep GM atrophy accounted for most of the regional atrophy seen in RRMS and SPMS. The areas of cortical and deep GM atrophy for each clinical subgroup, compared to controls, are summarised in Supplementary Material, Appendices 3 and 4.

### Subgroup comparison

At FWE 0.05, we detected only a small difference in MTR reduction in the SPMS versus RRMS subgroup comparison (124 voxels), but did not detect any significant differences in the other inter-subgroup comparisons for MTR reduction or atrophy alone, or co-localisation of both.

### Controls compared to all patients and clinical subtypes

Controls did not show any areas of consistent MTR reduction, atrophy, or co-localisation in GM compared to all patients, or to PPMS, SPMS and RRMS subgroups

### Re-analysis without FWE

**Figure 1.**
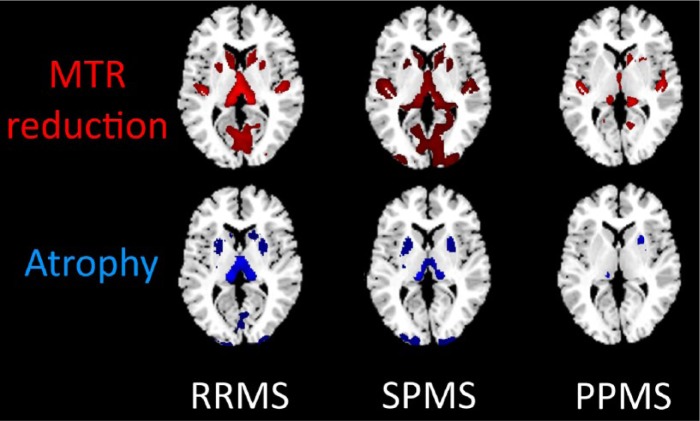
Voxel-based analysis (VBA) significant voxels (compared with controls) demonstrating magnetisation transfer ratio (MTR) reduction and atrophy by clinical subgroup (0.001, uncorrected). PPMS: primary progressive multiple sclerosis; RRMS: relapsing–remitting multiple sclerosis; SPMS: secondary progressive multiple sclerosis.

Given the small effect sizes obtained using FWE 0.05 for MS subgroups compared to controls, we reran the analysis without FWE, using an alpha level of 0.001, uncorrected. The results are shown in Table 3. Figure 1 illustrates the areas affected by MTR reduction and atrophy for each MS subgroup, compared to controls. We also reran the MS subgroup comparisons without FWE, using an alpha level of 0.001 uncorrected, and the summary of the findings are shown in Table 4. Supplementary Material, Appendices 5–9 show the GM areas affected.

**Table 3.** Voxel-based analysis (VBA) significant voxel counts (compared with controls) by clinical subgroup and location (0.001, uncorrected).

	Overall MTR reduction			Overall atrophy		
	RR	SP	PP	RR	SP	PP
**Total**	53,672	97,707	24,615	27,903	30,153	1857
**Cortex**	38,049	80,051	20,917	9562	17,647	712
**Deep GM**	15,623	17,656	3698	18,341	12,506	1145

GM: grey matter; MTR: magnetisation transfer ratio; PP: primary progressive; RR: relapsing–remitting; SP: secondary progressive.

**Table 4.** Voxel-based analysis (VBA) significant voxel counts of clinical subgroup comparisons by location (0.001, uncorrected).

	Overall MTR reduction						Overall atrophy					
	RR vs PP	RR vs SP	SP vs PP	SP vs RR	PP vs SP	PP vs RR	RR vs PP	RR vs SP	SP vs PP	SP vs RR	PP vs SP	PP vs RR
**Total**	4581	4808	8150	15,976	431	430	367	0	3886	939	0	0
**Cortex**	4580	4808	7321	15,951	431	430	329	0	3886	939	0	0
**Deep GM**	1	0	829	25	0	0	38	0	0	0	0	0

GM: grey matter; MTR: magnetisation transfer ratio; PP: primary progressive; RR: relapsing–remitting; SP: secondary progressive.

This suggests that in PPMS, there is considerably more MTR reduction than atrophy (24,615 voxels vs 1857 voxels respectively). SPMS shows the most widespread MTR reduction (97,707) and atrophy (30,153) of all the subgroups. The only difference seen compared to the analysis with FWE correction (*p*=0.05) is in RRMS, which revealed a greater number of voxels showing MTR reduction compared to atrophy (53,672 voxels vs 27,903 voxels).

Comparing MS subgroups (without FWE, using and alpha level of 0.001, uncorrected) reveals overall small differences in regions of MTR reduction and atrophy, and supports the findings from comparison with controls. Greater cortical MTR reduction and atrophy is seen in RRMS compared to PPMS, although PPMS subjects do also show MTR reduction in some cortical regions not seen in the RRMS subgroup. SPMS and RRMS comparisons reveal overall greater areas of MTR reduction and atrophy in the former, mainly in the cortex, although there are regions of MTR reduction and atrophy in RRMS not seen in the SPMS group. Similarly, when comparing SPMS and PPMS, while the latter does demonstrate greater MTR reduction in a few regions, overall SPMS subjects showed more MTR reduction and atrophy.

#### Co-localisation of MTR reduction and atrophy

Overlaying the areas of MTR reduction and atrophy for each MS subgroup allowed us to estimate which areas of the GM demonstrate co-localisation of these measures, albeit in a non-statistically tested manner. The results of these are included in Supplementary Material, Appendices 10–13.

### Correlation analysis using BPM

The results of the within-group correlation analysis of volume and MTR are summarised in Table 5.

**Table 5.** Number of voxels within each multiple sclerosis (MS) subgroup demonstrating positive correlation between magnetisation transfer ratio (MTR) and volume (family wise error (FWE) 0.05).

	RRMS	SPMS	PPMS
**Total GM voxels demonstrating either MTR or volume change**	101,273	137,994	31,514
**Total GM voxels demonstrating correlation between MTR and volume**	98,046	2706	2034
**Cortical GM voxels**	85,599	2626	2030
**Deep GM voxels**	12,447	80	4

GM: grey matter; PP: primary progressive; RR: relapsing–remitting; SP: secondary progressive.

SPMS patients show the greatest number of GM voxels demonstrating either MTR reduction or atrophy, but only a small proportion of these voxels have significant correlation between the two. RRMS patients also show a large number of GM voxels demonstrating either abnormality, the majority of which significantly correlate. In PPMS patients, there are less GM voxels overall demonstrating either abnormality, but only a small proportion of these correlate significantly. In all MS subgroups, there are more voxels demonstrating positive correlation between MTR and volume in the cortex compared to the deep GM. RRMS patients show the greatest number of correlating voxels overall in the GM, as well as proportionately greatest deep GM involvement. In SPMS and PPMS patients there are far fewer positively correlating voxels overall, the vast majority of which are in the cortex.

## Discussion

MTR reduction is thought to reflect demyelination, as shown by histopathological work in cortical GM,^19^ as well as WM,^18^ and atrophy is predominantly secondary to neuro-axonal loss.^11^ Co-localisation of MTR reduction and atrophy would therefore suggest a link between demyelination and neuro-axonal loss. The presence of atrophy alone suggests neuro-axonal loss without demyelination. The presence of MTR reduction alone without significant atrophy co-localisation is suggestive of demyelination without significant neuronal loss.

In this study, the results reveal differences in the spatial patterns of MTR reduction and atrophy in the MS cohort overall and between different clinical subgroups that may help improve our understanding of the pathophysiological basis of GM pathology in MS.

In all MS subgroups, MTR reduction was more extensive than atrophy. Although absolute numbers of voxels demonstrating cortical MTR reduction was higher than in the deep GM, taking into consideration the mean voxel counts for cortical GM and deep GM in each group (Table 1), a greater proportion of the deep GM compartment showed MTR reduction compared with the cortex, in all subgroups, but especially in RRMS and SPMS subgroups. Atrophy was seen in both RRMS and SPMS subgroups, and both showed greater involvement of the deep GM than the cortex, relative to the respective sizes of the compartments.

Within-group correlation analysis suggests that in RRMS, there more abnormal voxels overall demonstrating either MTR reduction or atrophy, and most of these demonstrating significant correlation between the two, with the deep GM showing especially high proportional involvement. In SPMS and PPMS the number of correlating voxels is less overall, with proportionately fewer correlating voxels in the deep GM.

These results suggest that there is significant neuro-axonal loss, especially in the deep GM of the relapse-onset groups (RRMS and SPMS). A previous VBM study of atrophy in the GM of early PPMS patients (within five years of symptom onset) demonstrated a shift in the pattern of atrophy as the disease progressed, with deep GM atrophy seen early on and greater involvement of the cortex later.^36^ In the progressive groups (SPMS and PPMS), there is proportionately greater cortical demyelination, which is agreement with histopathological work.^3,37^ Further, this appears to be relatively greater in SPMS compared with PPMS; cortical demyelination has recently been linked with meningeal inflammation, and in turn this is more evident in SPMS than PPMS.^8,9^

RRMS shows the greatest number of voxels demonstrating correlation between MTR reduction and atrophy in the deep GM, which is consistent with previous histopathological work on relapse-onset MS.^5^ This suggests that, in the deep GM of this subgroup, demyelination and neuro-axonal loss may be linked. For instance, inflammatory demyelination in the deep GM may lead to secondary neuro-axonal loss, or perhaps primary neurodegenerative neuro-axonal loss in the deep GM may lead to secondary loss of myelin. In SPMS patients, while there is increased demyelination and neuro-axonal loss overall, only a small amount of this co-exists in the same regions, especially in the deep GM. In PPMS patients, deep GM demyelination and neuro-axonal loss is seen, but to a lesser than in RRMS and SPMS patients, and correlation is minimal. This suggests that in progressive MS the two pathologies are, for the most part, not due to a common process.

Although significant inflammation is usually seen in cortical demyelinating lesions in early MS, this is usually not so in MS of a longer duration.^38–40^ A recently published histopathological study suggested that the deep GM showed inflammation that is less than in the white matter but more than in the cortical GM.^41^ As demyelination usually occurs within the context of inflammation, this may explain why, in our study, the deep GM compartment showed relatively greater amounts of demyelination compared to the cortex in all MS subgroups, proportional to the respective compartment sizes. Our results also suggest that inflammatory demyelination plays a relatively limited direct role in neuro-axonal loss, especially in the cortex of progressive MS. Atrophy may thus be either a primary neurodegenerative process, or may be secondary to the downstream effects of WM inflammatory pathology without associated local demyelination.^1^

Our results need to be interpreted with respect to the methodology used. This was a cross-sectional study with a modest sample size, which may affect the generalisability of the findings. In PPMS patients the relatively small voxel counts overall may be a reflection of the relatively fewer subjects in this cohort. The use of FWE (*p*=0.05) produced results with small effect sizes, and this is likely a reflection of the conservative nature of the VBA analysis (which detects consistent group level changes rather than any voxel that is abnormal in any individual subject). For the MTR analysis we also used a threshold of 90% for GM extraction to limit partial volume effects. However, given that a substantial proportion of cortical GM demyelination occurs in the subpial layers,^3,9^ this may also have reduced the sensitivity to cortical demyelination. We also used a tissue segmentation pipeline optimised for MS, with lesion-filling prior to processing by SPM8, and custom built DARTEL templates, which have been shown to reduce artifactual changes in MS studies,^42^ but may also further reduce effect size. Using an uncorrected statistical threshold (*p*=0.001), results of the comparisons with controls were broadly consistent with those from the more stringent FWE (*p*=0.05) analysis, but with greater effect sizes, confirming that the choice of threshold does not substantially alter our main conclusions.

## Conclusion

The results of this study suggest, that in RRMS patients, demyelination and neuro-axonal loss often occur in the same regions in the deep GM, implying that these two processes may be linked in this GM compartment. In progressive MS patients, co-existing demyelination and neuro-axonal loss was less commonly seen, which argues against these processes being linked. Overall, this study argues against a single underlying mechanism as a cause of GM pathology in MS clinical subgroups.

## Supplementary Material

Supplementary material
