# Supplementary material for: Regional patterns of grey matter atrophy and magnetisation transfer ratio abnormalities in multiple sclerosis clinical subgroups: A voxel-based analysis study
Source: Mult Scler. 2015 Apr;21(4):423–32. doi: 10.1177/1352458514546513 (PMC4390521; doi:10.1177/1352458514546513)
Supplement: Supplementary material [file MSJ_546513_supplementary_data.docx]

| Appendix 1: Cortical MTR reduction (FWE 0.05) | | | | | | | | | | | | | | |
| --- | --- | --- | --- | --- | --- | --- | --- | --- | --- | --- | --- | --- | --- | --- |
| *RRMS* | | | | | ***SPMS*** | | | | | ***PPMS*** | | | | |
| *Area* | *Voxels* | *X* | *Y* | *Z* | *Area* | *Voxels* | *X* | *Y* | *Z* | *Area* | *Voxels* | *X* | *Y* | *Z* |
| Heschl L | 90 | -41 | -22 | 8 | Precentral L | 1795 | -31 | -19 | 66 | Heschl R | 85 | 48 | -15 | 5 |
| Temporal Sup L | 58 | -42 | -23 | 8 | Postcentral L | 1411 | -49 | -10 | 39 | Temporal Sup R | 76 | 48 | -15 | 4 |
| Precentral R | 45 | 37 | -15 | 49 | Temporal Sup L | 1275 | -41 | -25 | 13 | Precentral R | 59 | 41 | -10 | 37 |
| Postcentral L | 38 | -40 | -17 | 43 | Postcentral R | 1019 | 45 | -11 | 3 | Temporal Sup L | 45 | -44 | -23 | 7 |
| Lingual R | 37 | 9 | -32 | 1 | Precentral R | 781 | 43 | -11 | 7 | Postcentral R | 26 | 41 | -10 | 36 |
| Heschl R | 33 | 46 | -20 | 7 | Heschl L | 649 | -39 | -26 | 1 | Lingual R | 10 | 15 | -35 | 0 |
| Hippocampus L | 28 | -13 | -32 | 9 | Calcarine R | 579 | 13 | -91 | 7 | Precuneus R | 10 | 13 | -35 | 3 |
| Temporal Sup R | 28 | 47 | -20 | 7 | Heschl R | 459 | 41 | -24 | -6 | Hippocampus R | 7 | 17 | -34 | 1 |
| Hippocampus R | 18 | 11 | -32 | 9 | Temporal Sup R | 280 | 43 | -24 | -2 |  |  |  |  |  |
| Lingual L | 8 | -9 | -34 | 3 | Lingual L | 174 | -10 | -33 | 0 |  |  |  |  |  |
| Precuneus R | 8 | 13 | -34 | 4 | Lingual R | 165 | 10 | -33 | 1 |  |  |  |  |  |
| Postcentral R | 6 | 43 | -9 | 36 | Paracentral Lobule L | 142 | -6 | -18 | -4 |  |  |  |  |  |
| Cingulum Post R | 2 | 11 | -34 | 7 | Occipital Inf R | 124 | 31 | -95 | -4 |  |  |  |  |  |
|  |  |  |  |  | Rolandic Oper L | 91 | -39 | -28 | -6 |  |  |  |  |  |
|  |  |  |  |  | Occipital Mid L | 89 | -29 | -96 | 36 |  |  |  |  |  |
|  |  |  |  |  | Hippocampus L | 87 | -13 | -34 | 38 |  |  |  |  |  |
|  |  |  |  |  | Hippocampus R | 71 | 13 | -34 | 2 |  |  |  |  |  |
|  |  |  |  |  | Precuneus R | 51 | 13 | -34 | 3 |  |  |  |  |  |
|  |  |  |  |  | ParaHippocampal L | 33 | -18 | -32 | 73 |  |  |  |  |  |
|  |  |  |  |  | Precuneus L | 18 | -12 | -36 | 11 |  |  |  |  |  |
|  |  |  |  |  | Occipital Inf L | 12 | -29 | -95 | 11 |  |  |  |  |  |
|  |  |  |  |  | Insula L | 10 | -46 | -12 | 10 |  |  |  |  |  |
|  |  |  |  |  | Cingulum Post R | 5 | 11 | -34 | 11 |  |  |  |  |  |

| Appendix 2: Deep GM MTR reduction (FWE 0.05) | | | | | | | | | | | | | | |
| --- | --- | --- | --- | --- | --- | --- | --- | --- | --- | --- | --- | --- | --- | --- |
| *RRMS* | | | | | ***SPMS*** | | | | | ***PPMS*** | | | | |
| *Area* | *Voxels* | *X* | *Y* | *Z* | *Area* | *Voxels* | *X* | *Y* | *Z* | *Area* | *Voxels* | *X* | *Y* | *Z* |
| Thalamus L | 1631 | -10 | -28 | 10 | Thalamus L | 1327 | -10 | -31 | 5 |  |  |  |  |  |
| Thalamus R | 1411 | 2 | -21 | 4 | Caudate R | 1021 | 12 | 1 | 17 |  |  |  |  |  |
| Caudate R | 95 | 12 | 4 | 17 | Thalamus R | 998 | 10 | -31 | 4 |  |  |  |  |  |
| Caudate L | 79 | -20 | -24 | 21 | Caudate L | 443 | -14 | 1 | 19 |  |  |  |  |  |

| Appendix 3: Cortical Atrophy (FWE 0.05) | | | | | | | | | | | | | | |
| --- | --- | --- | --- | --- | --- | --- | --- | --- | --- | --- | --- | --- | --- | --- |
| *RRMS* | | | | | ***SPMS*** | | | | | ***PPMS*** | | | | |
| *Area* | *Voxels* | *X* | *Y* | *Z* | *Area* | *Voxels* | *X* | *Y* | *Z* | *Area* | *Voxels* | *X* | *Y* | *Z* |
| Precentral L | 162 | -33 | -21 | 60 | Precentral L | 111 | -36 | -21 | 65 |  |  |  |  |  |
| Hippocampus L | 24 | -13 | -34 | 3 | Occipital Mid L | 6 | -30 | -97 | -3 |  |  |  |  |  |
| Lingual L | 20 | -9 | -34 | 3 |  |  |  |  |  |  |  |  |  |  |

| Appendix 4: Deep GM Atrophy (FWE 0.05) | | | | | | | | | | | | | | |
| --- | --- | --- | --- | --- | --- | --- | --- | --- | --- | --- | --- | --- | --- | --- |
| *RRMS* | | | | | ***SPMS*** | | | | | ***PPMS*** | | | | |
| *Area* | *Voxels* | *X* | *Y* | *Z* | *Area* | *Voxels* | *X* | *Y* | *Z* | *Area* | *Voxels* | *X* | *Y* | *Z* |
| Thalamus R | 2714 | 3 | -10 | 7 | Thalamus L | 807 | -14 | -26 | 3 |  |  |  |  |  |
| Thalamus L | 2033 | -12 | -28 | 5 | Thalamus R | 614 | 9 | -12 | 7 |  |  |  |  |  |
| Putamen L | 71 | -29 | -12 | 0 |  |  |  |  |  |  |  |  |  |  |
| Pallidum L | 2 | -27 | -9 | -2 |  |  |  |  |  |  |  |  |  |  |

| Appendix 5: MTR reduction RRMS subgroup comparisons (0.001, uncorrected) | | | | | | | | | |
| --- | --- | --- | --- | --- | --- | --- | --- | --- | --- |
| *RRMS vs PPMS* | | | | | ***RRMS vs SPMS*** | | | | |
| *Area* | *Voxels* | *X* | *Y* | *Z* | *Area* | *Voxels* | *X* | *Y* | *Z* |
| *Cortical GM* | | | | | *Cortical GM* | | | | |
| Cingulum Mid R | 2351 | 1 | -26 | 36 | Precuneus R | 2386 | 2 | -42 | 56 |
| Cingulum Mid L | 1176 | 0 | -26 | 36 | Cingulum Mid L | 869 | -5 | -43 | 53 |
| Precuneus R | 198 | 6 | -50 | 29 | Precuneus L | 691 | -5 | -44 | 54 |
| Cingulum Post R | 129 | 6 | -49 | 30 | Cingulum Mid R | 429 | 5 | -41 | 52 |
| Supp Motor Area R | 119 | 7 | -21 | 49 | Frontal Mid R | 100 | 30 | 9 | 55 |
| Paracentral Lobule L | 119 | -3 | -32 | 52 | Frontal Mid L | 79 | -31 | 29 | 36 |
| Occipital Mid R | 118 | 38 | -82 | 7 | Cingulum Post L | 56 | 0 | -52 | 34 |
| Temporal Inf R | 92 | 55 | -55 | -8 | Occipital Sup R | 56 | 26 | -62 | 41 |
| Temporal Inf L | 87 | -41 | -4 | -39 | Frontal Sup R | 36 | 27 | 1 | 65 |
| Precentral R | 79 | 40 | -1 | 47 | Cingulum Post R | 32 | 1 | -51 | 32 |
| ParaHippocampal L | 39 | -24 | -6 | -34 | Paracentral Lobule R | 25 | 4 | -43 | 61 |
| Fusiform L | 26 | -25 | -7 | -34 | Angular R | 13 | 28 | -62 | 42 |
| Lingual R | 9 | 16 | -89 | -11 | Frontal Sup L | 12 | -17 | 27 | 52 |
| SupraMarginal L | 8 | -56 | -42 | 36 | Parietal Inf R | 9 | 42 | -45 | 45 |
| Frontal Inf Orb L | 7 | -46 | 27 | -10 | Parietal Sup R | 6 | 24 | -52 | 63 |
| Cingulum Post L | 6 | -1 | -31 | 34 | Supp Motor Area L | 3 | 0 | -1 | 65 |
| Paracentral Lobule R | 6 | 6 | -31 | 52 | Supp Motor Area R | 3 | 1 | 0 | 65 |
| Lingual L | 4 | -21 | -79 | -12 | Paracentral Lobule L | 3 | -1 | -37 | 55 |
| Parietal Inf R | 3 | 38 | -46 | 43 |  |  |  |  |  |
| Parietal Inf L | 2 | -56 | -42 | 37 |  |  |  |  |  |
| Occipital Inf L | 1 | -48 | -59 | -15 |  |  |  |  |  |
| Temporal Sup L | 1 | -57 | -5 | -2 |  |  |  |  |  |
|  | | | | |  | | | | |
| *Deep GM* | | | | | *Deep GM* | | | | |
| Putamen L | 1 | -29 | 3 | 9 | No significant differences detected | | | | |

| Appendix 6: MTR reduction PPMS subgroup comparisons (0.001, uncorrected) | | | | | | | | | |
| --- | --- | --- | --- | --- | --- | --- | --- | --- | --- |
| *PPMS vs SPMS* | | | | | ***PPMS vs RRMS*** | | | | |
| *Area* | *Voxels* | *X* | *Y* | *Z* | *Area* | *Voxels* | *X* | *Y* | *Z* |
| *Cortical GM* | | | | | *Cortical GM* | | | | |
| Frontal Mid L | 212 | -32 | 29 | 34 | Frontal Inf Oper R | 336 | 52 | 19 | 39 |
| Frontal Mid R | 136 | 35 | 23 | 41 | Frontal Mid R | 45 | 53 | 19 | 41 |
| SupraMarginal R | 61 | 53 | -33 | 39 | Frontal Inf Orb R | 25 | 53 | 21 | -2 |
| Precentral L | 10 | -48 | 11 | 50 | Temporal Pole Sup R | 9 | 53 | 19 | -4 |
| Frontal Sup L | 8 | -20 | 18 | 62 | Frontal Inf Tri R | 4 | 53 | 21 | 1 |
| Frontal Inf Oper L | 2 | -50 | 20 | 34 |  |  |  |  |  |
| Parietal Inf L | 2 | -45 | -48 | 50 |  |  |  |  |  |
|  | | | | |  | | | | |
| *Deep GM* | | | | | *Deep GM* | | | | |
| No significant differences detected | | | | |  | | | | |

| Appendix 7: MTR reduction SPMS subgroup comparisons (0.001, uncorrected) | | | | | | | | | |
| --- | --- | --- | --- | --- | --- | --- | --- | --- | --- |
| *SPMS vs PPMS* | | | | | ***SPMS vs RRMS*** | | | | |
| *Area* | *Voxels* | *X* | *Y* | *Z* | *Area* | *Voxels* | *X* | *Y* | *Z* |
| *Cortical GM* | | | | | *Cortical GM* | | | | |
| Calcarine R | 1687 | 14 | -92 | -4 | Calcarine R | 4717 | 21 | -48 | 8 |
| Occipital Inf R | 828 | 36 | -94 | -4 | Lingual R | 1957 | 10 | -78 | -1 |
| Precentral R | 812 | 47 | -10 | 41 | Precentral L | 1904 | -28 | -19 | 69 |
| Precentral L | 736 | -33 | -20 | 65 | Temporal Sup L | 1377 | -56 | -14 | 7 |
| Paracentral Lobule L | 456 | -4 | -16 | 76 | Precentral R | 1329 | 30 | -20 | 71 |
| Lingual R | 452 | 14 | -92 | -6 | Hippocampus R | 506 | 18 | -35 | 6 |
| Occipital Mid L | 366 | -19 | -84 | 18 | Postcentral L | 432 | -53 | -4 | 24 |
| Cingulum Mid R | 363 | 13 | -17 | 45 | Precuneus R | 410 | 25 | -51 | 3 |
| Occipital Mid R | 269 | 24 | -94 | 9 | Occipital Mid L | 394 | -20 | -83 | 15 |
| Postcentral L | 261 | -47 | -7 | 36 | Postcentral R | 285 | 47 | -9 | 40 |
| Postcentral R | 236 | 48 | -9 | 40 | Temporal Pole Sup L | 244 | -45 | 14 | -14 |
| Occipital Sup L | 191 | -19 | -83 | 18 | Heschl L | 239 | -48 | -16 | 7 |
| Lingual L | 151 | -19 | -80 | -7 | Occipital Inf L | 195 | -35 | -80 | -3 |
| Supp Motor Area R | 112 | 10 | -19 | 49 | Hippocampus L | 185 | -19 | -31 | -5 |
| Temporal Pole Sup L | 84 | -45 | 22 | -16 | ParaHippocampal L | 149 | -19 | -33 | -6 |
| Calcarine L | 83 | -19 | -75 | 7 | Calcarine L | 148 | 4 | -83 | 3 |
| Occipital Inf L | 72 | -35 | -80 | -2 | ParaHippocampal R | 132 | 21 | -30 | -9 |
| Occipital Sup R | 54 | 23 | -94 | 11 | Frontal Inf Orb R | 116 | 52 | 19 | -6 |
| Cuneus R | 23 | 15 | -92 | 7 | Temporal Mid L | 79 | -48 | -10 | -14 |
| Fusiform L | 21 | -20 | -81 | -9 | Occipital Sup L | 74 | -19 | -83 | 15 |
| Supp Motor Area L | 7 | -3 | -11 | 76 | Insula L | 66 | -46 | -12 | 3 |
| Paracentral Lobule R | 6 | 9 | -28 | 53 | Temporal Pole Sup R | 54 | 53 | 19 | -6 |
| Temporal Sup L | 3 | -54 | -12 | -3 | Occipital Inf R | 53 | 25 | -88 | -5 |
| Hippocampus L | 2 | -21 | -30 | -6 | Temporal Sup R | 36 | 47 | -10 | 2 |
|  |  |  |  |  | Lingual L | 29 | -16 | -34 | -3 |
|  |  |  |  |  | Frontal Inf Oper R | 10 | 54 | 22 | -1 |
|  |  |  |  |  | Cuneus R | 8 | 7 | -82 | 15 |
|  |  |  |  |  | Frontal Inf Tri R | 7 | 55 | 22 | 0 |
|  |  |  |  |  | Fusiform R | 7 | 27 | -87 | -5 |
|  |  |  |  |  | Frontal Sup Orb R | 6 | 13 | 48 | -24 |
|  |  |  |  |  | Insula R | 5 | 46 | -6 | -3 |
|  |  |  |  |  | Rolandic Oper L | 2 | -48 | -7 | 2 |
|  |  |  |  |  | Frontal Inf Orb L | 1 | -46 | 17 | -12 |
|  |  |  |  |  | Olfactory R | 1 | 4 | 17 | -1 |
|  |  |  |  |  | Cingulum Mid R | 1 | 14 | -15 | 42 |
|  | | | | |  | | | | |
| *Deep GM* | | | | | *Deep GM* | | | | |
| Thalamus L | 546 | -7 | -27 | 3 | Thalamus R | 15 | 16 | -33 | 6 |
| Thalamus R | 283 | 5 | -21 | 0 | Caudate L | 6 | -7 | 22 | -2 |
|  |  |  |  |  | Caudate R | 4 | 4 | 16 | -1 |

| Appendix 8: Atrophy RRMS subgroup comparisons (0.001, uncorrected) | | | | | | | | | |
| --- | --- | --- | --- | --- | --- | --- | --- | --- | --- |
| *RRMS vs PPMS* | | | | | ***RRMS vs SPMS*** | | | | |
| *Area* | *Voxels* | *X* | *Y* | *Z* | *Area* | *Voxels* | *X* | *Y* | *Z* |
| *Cortical GM* | | | | | *Cortical GM* | | | | |
| Frontal Mid Orb R | 91 | 33 | 49 | -3 | No significant differences detected | | | | |
| Lingual L | 53 | -20 | -81 | -12 |  |  |  |  |  |
| Fusiform L | 46 | -21 | -83 | -6 |  |  |  |  |  |
| Occipital Mid L | 6 | -20 | -85 | -4 |  |  |  |  |  |
| Frontal Mid R | 4 | 32 | 49 | 0 |  |  |  |  |  |
| Insula R | 4 | 28 | 22 | -16 |  |  |  |  |  |
|  |  |  |  |  |  |  |  |  |  |
| *Deep GM* | | | | | *Deep GM* | | | | |
| Thalamus L | 17 | -15 | -12 | 17 | No significant differences detected | | | | |
| Caudate R | 14 | 11 | 6 | 8 |  | | | | |
| Pallidum R | 4 | 15 | 6 | 6 |  | | | | |
| Caudate L | 3 | -14 | -11 | 21 |  | | | | |

| Appendix 9: Atrophy SPMS subgroup comparisons (0.001, uncorrected) | | | | | | | | | |
| --- | --- | --- | --- | --- | --- | --- | --- | --- | --- |
| *SPMS vs PPMS* | | | | | ***SPMS vs RRMS*** | | | | |
| *Area* | *Voxels* | *X* | *Y* | *Z* | *Area* | *Voxels* | *X* | *Y* | *Z* |
| *Cortical GM* | | | | | *Cortical GM* | | | | |
| Frontal Sup R | 1490 | 17 | 33 | 44 | Occipital Mid L | 302 | -24 | -86 | 14 |
| Supp Motor Area R | 740 | 10 | 12 | 62 | Precentral L | 254 | -36 | -21 | 68 |
| Temporal Inf R | 450 | 53 | -49 | -24 | Postcentral L | 199 | -63 | -8 | 24 |
| Frontal Mid L | 332 | -31 | 27 | 35 | Precentral R | 124 | 33 | -18 | 61 |
| Temporal Mid L | 267 | -43 | 3 | -29 | Precuneus R | 32 | 17 | -63 | 27 |
| SupraMarginal L | 192 | -52 | -51 | 25 | Calcarine L | 24 | -10 | -102 | -8 |
| Frontal Sup Medial R | 146 | 12 | 34 | 49 | Cuneus R | 2 | 19 | -63 | 29 |
| Angular L | 92 | -51 | -51 | 26 | Occipital Inf L | 2 | -10 | -101 | -7 |
| ParaHippocampal L | 71 | -14 | 5 | -26 |  |  |  |  |  |
| Parietal Inf L | 42 | -44 | -49 | 46 |  |  |  |  |  |
| Temporal Mid R | 28 | 51 | -61 | 1 |  |  |  |  |  |
| Precuneus L | 20 | -12 | -48 | 50 |  |  |  |  |  |
| Temporal Inf L | 12 | -43 | 3 | -30 |  |  |  |  |  |
| Parietal Inf R | 2 | 44 | -57 | 47 |  |  |  |  |  |
| Calcarine R | 1 | 15 | -84 | 15 |  |  |  |  |  |
|  |  |  |  |  |  |  |  |  |  |
| *Deep GM* | | | | | *Deep GM* | | | | |
| Putamen L | 1 | -29 | 3 | 9 | No significant differences detected | | | | |

| Appendix 10: VBA significant voxel counts (compared with controls) by clinical subgroup and location (FWE 0.05) | | | | |
| --- | --- | --- | --- | --- |
|  | MTR reduction and Atrophy | | |  |
|  | RR | SP | PP |  |
| Total | 2157 | 610 | 0 |  |
| Cortex | 21 | 109 | 0 |  |
| Deep GM | 2136 | 501 | 0 |  |
| RR: Relapsing Remitting; SP: Secondary Progressive; PP: Primary Progressive; MTR: Magnetisation Transfer Ratio; VBA: Voxel Based Analysis; FWE: Family Wise Error; GM: Grey Matter | | | | |

| Appendix 11: VBA significant voxel counts (compared with controls) by clinical subgroup and location (0.001, uncorrected) | | | | |
| --- | --- | --- | --- | --- |
|  | MTR reduction and Atrophy | | |  |
|  | RR | SP | PP |  |
| Total | 14649 | 18867 | 875 |  |
| Cortex | 4770 | 11985 | 703 |  |
| Deep GM | 9879 | 6882 | 172 |  |
| RR: Relapsing Remitting; SP: Secondary Progressive; PP: Primary Progressive; MTR: Magnetisation Transfer Ratio; VBA: Voxel Based Analysis; FWE: Family Wise Error; GM: Grey Matter | | | | |

| Appendix 12: Cortical MTR reduction and Atrophy Co-localisation (FWE 0.05) | | | | | | | | | | | | | | |
| --- | --- | --- | --- | --- | --- | --- | --- | --- | --- | --- | --- | --- | --- | --- |
| *RRMS* | | | | | ***SPMS*** | | | | | ***PPMS*** | | | | |
| *Area* | *Voxels* | *X* | *Y* | *Z* | *Area* | *Voxels* | *X* | *Y* | *Z* | *Area* | *Voxels* | *X* | *Y* | *Z* |
| Hippocampus L | 15 | -13 | -34 | 5 | Precentral L | 103 | -33 | -19 | 65 |  |  |  |  |  |
| Lingual L | 6 | -9 | -34 | 3 | Occipital Mid L | 6 | -30 | -97 | -3 |  |  |  |  |  |

| Appendix 13: Deep GM MTR reduction and Atrophy Co-localisation (FWE 0.05) | | | | | | | | | | | | | | |
| --- | --- | --- | --- | --- | --- | --- | --- | --- | --- | --- | --- | --- | --- | --- |
| *RRMS* | | | | | ***SPMS*** | | | | | ***PPMS*** | | | | |
| *Area* | *Voxels* | *X* | *Y* | *Z* | *Area* | *Voxels* | *X* | *Y* | *Z* | *Area* | *Voxels* | *X* | *Y* | *Z* |
| Thalamus L | 1143 | -10 | -28 | 10 | Thalamus L | 500 | -10 | -31 | 5 |  |  |  |  |  |
| Thalamus R | 993 | 1 | -20 | 5 | Thalamus R | 1 | 13 | -28 | 2 |  |  |  |  |  |
